# Complete chloroplast genome sequence of a bulbous flowering plant, *Allium triquetrum* Linnaeus, 1753 (Amaryllidaceae)

**DOI:** 10.1080/23802359.2022.2135402

**Published:** 2022-11-02

**Authors:** Xiaoxiao Min, Peiling Li, Shoufu Gong, Qingsong Zhu

**Affiliations:** College of Horticulture, Xinyang Agricultural and Forestry University, Xinyang, China

**Keywords:** *Allium triquetrum*, chloroplast genome, phylogenetic analysis

## Abstract

*Allium triquetrum* (Linnaeus, 1753) is a bulbous flowering plant of the genus *Allium* (Amaryllidaceae), native to the Mediterranean basin, and is now widespread and invasive in different parts of the world via ornamental horticultural trade. However, to date, the genomic study of *A. triquetrum* has lagged, which impedes the development of appropriate utilization and management practices for this species. Here, we report the complete chloroplast genome sequence of *A. triquetrum*. The chloroplast genome size of *A. triquetrum* was 153,298 bp, consisting of a pair of inverted repeat regions (26,547 bp), separated by a large single-copy (82,875 bp) region and a small single-copy (17,329 bp) region. Genome annotation predicted 133 genes, including 87 protein-coding genes, 38 tRNA genes, and eight rRNA genes. Phylogenetic analysis based on 60 whole chloroplast genome sequences of *Allium* species suggested that *A. triquetrum* and *A. moly* are sister to each other along with the clade of *A. fasciculatum*, *A. hookeri*, and *A. macranthum*.

*Allium triquetrum* Linnaeus, 1753 (Amaryllidaceae; also known as three-cornered leek) is a bulbiferous monocotyledonous herb native to the central and western Mediterranean regions of Eurasia (Tehranchian et al. 2014). Not only is this species valued as ornamental for its flowers, but it can also be traditionally used in folk medicine, especially for wound healing (Rabah et al. 2020). Due to anthropogenic propagule dispersal via the ornamental horticulture trade, followed by ‘escape’ into natural environments, this species has been invasive in different parts of the world (Lesieur et al. 2018, 2022). However, to date, no genomic studies have reported on *A. triquetrum*, which impedes the development of its utilization and management practices (Lesieur et al. 2022). Here, we report the chloroplast genome sequence of *A. triquetrum* and reconstruct its phylogenetic relationship with other *Allium* species.

Fresh *A. triquetrum* leaves were sampled from the Nanjing Botanical Garden, Mem. Sun Yat-sen (E118°83′, N32°06′) with special permission from Xinyang Agricultural and Forestry University. Voucher specimens and DNA were deposited at the College of Horticulture, Xinyang Agricultural and Forestry University (https://www.xyafu.edu.cn/, contact Xiaoxiao Min, minxiaoxiao2158@126.com) under voucher number FKDO210424906. DNA was extracted using DNA Plantzol Reagent (Invitrogen Trading Co., Ltd., Shanghai, China). DNA library construction and 150-bp paired-end sequencing were performed using the Illumina NovaSeq 6000 platform (Novogene, Tianjin, China). The chloroplast genome was assembled using the GetOrganelle pipeline (Jin et al. 2020), and annotated using Geneious Prime v.2021.1.1 (http://www.geneious.com) using *A. kingdonii* (MK294559) as a reference. The annotated chloroplast genome sequence of *A. triquetrum* has been deposited in GenBank (accession number: ON310895).

The chloroplast genome sequence of *A. triquetrum* was 153,298 bp in length and exhibited a typical quadripartite structure, consisting of a pair of inverted repeat regions (IRs) of 26,547 bp, separated by a large single-copy (LSC) region of 82,875 bp and a small single-copy (SSC) region of 17,329 bp. The chloroplast genome encoded 133 genes, of which 111 (77 protein-coding genes, 30 tRNA genes, and four rRNA genes) were unique and 22 (10 protein-coding genes, eight tRNA genes, and four rRNA genes) were duplicated in the IRs. The gene *rps12* was trans-spliced with three exons: C-terminal exons 2 and 3 were located in the IRs, while exon 1 was ∼28.1 kilobase pairs downstream of the nearest copy of exons 2 and 3, and ∼69.8 kilobase pairs away from the distal copy of exons 2 and 3. The overall GC content was 36.8%, whereas the GC contents in IR, LSC, and SSC were 42.5%, 34.6%, and 29.9%, respectively.

The phylogenetic relationship of *Allium* was reconstructed using the maximum-likelihood (ML) method based on the multiple alignment of *A. triquetrum* and 59 previously reported chloroplast genomes of *Allium*, with *Agapanthus coddii* (KX790363) and *Narcissus poeticus* (MH706763) as outgroups. ML analysis was conducted using RAxML-HPC v.8.2.8 (Stamatakis 2014) with 1000 bootstrap replicates on the CIPRES Science Gateway (https://www.phylo.org/). The phylogenetic topology strongly supported that *A. triquetrum* and *A. moly* were sister to each other, and jointly sister to the clade of *A. fasciculatum*, *A. hookeri*, and *A. macranthum* (Figure 1).

**Figure 1.**
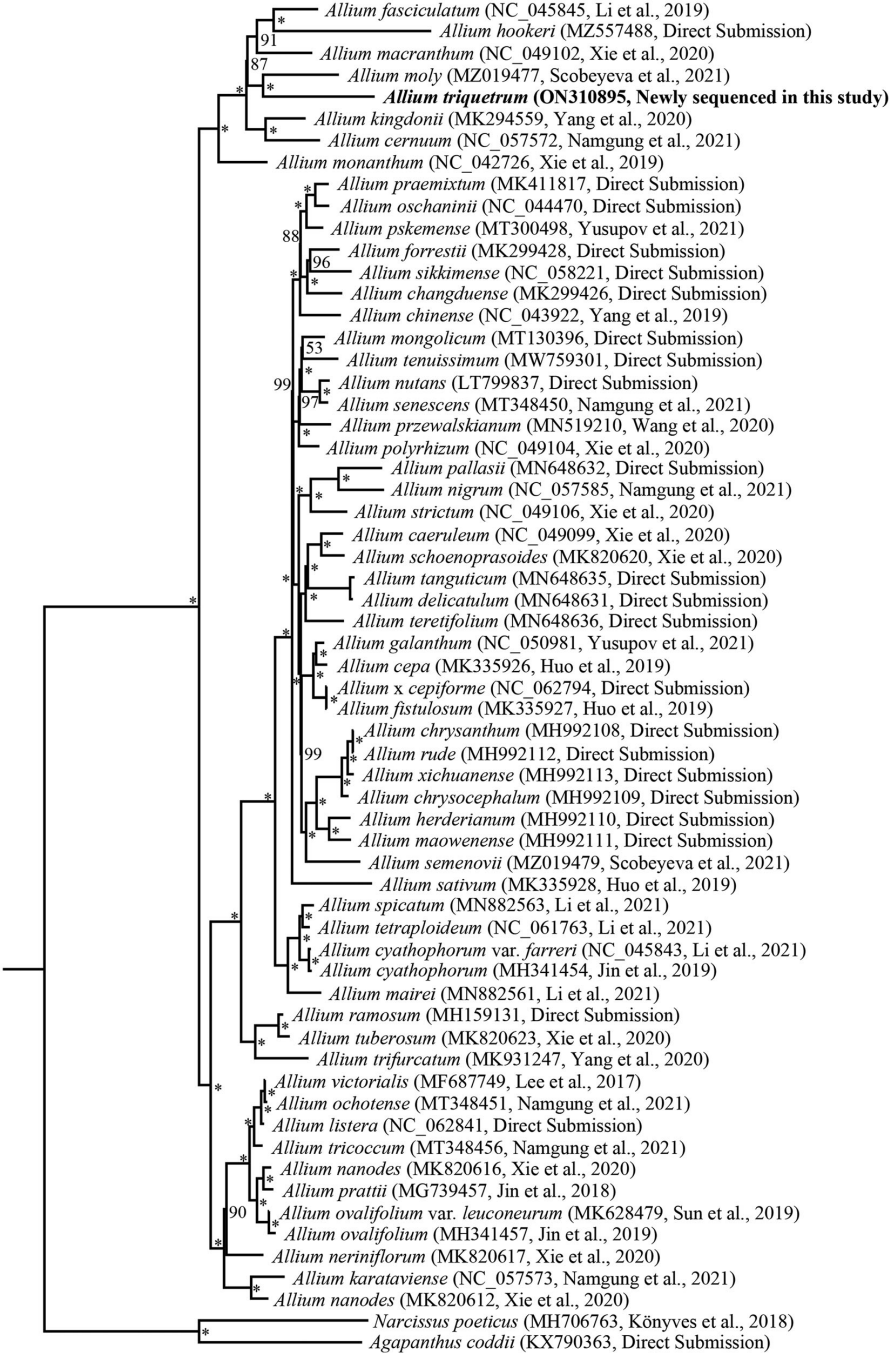
Phylogenetic tree inferred by maximum-likelihood (ML) method based on complete chloroplast genomes of 60 *Allium* species with *Agapanthus coddii* (KX790363) and *Narcissus poeticus* (MH706763) as outgroups. Numbers near the nodes represent ML bootstrap values. Bootstrap values of 100% are marked at the branch points with an asterisk (*).

## Author contributions

ZQ and GS conceived the project. MX and LP collected samples and analyzed data. MX wrote the manuscript. ZQ and LP revised the manuscript. All authors read and approved the manuscript.

## Data Availability

The genome sequence data that support the findings of this study are openly available in GenBank of NCBI at https://www.ncbi.nlm.nih.gov under the accession no. ON310895. The associated BioProject, SRA, and Bio-Sample numbers are PRJNA825297, SRR18706756, and SAMN27511544, respectively.
